# Systemic IgG responses to glycosylated mucinase YghJ after experimental enterotoxigenic *Escherichia coli* infection

**DOI:** 10.1186/s13099-025-00748-7

**Published:** 2025-09-25

**Authors:** Saman Riaz, Hans Steinsland, Anders Boysen, Kurt Hanevik

**Affiliations:** 1 Department of Clinical Science, University of Bergen, Bergen, Norway; 2 Centre for Intervention Science in Maternal and Child Health (CISMAC), Centre for International Health, Department of Global Public Health and Primary Care, University of Bergen, Bergen, Norway; 3 Department of Biomedicine, University of Bergen, Bergen, Norway; 4 GlyProVac ApS, Rørhatten 4, Odense, Denmark; 5 Department of Medicine, National Centre for Tropical Infectious Diseases, Haukeland University Hospital, Bergen, Norway

**Keywords:** IgG, YghJ, SslE, Enterotoxigenic, *Escherichia coli*, Glycosylated epitopes, Glycosylation, Vaccine antigen

## Abstract

**Background:**

The availability of a broadly protective vaccine against pathogenic *Escherichia coli* could help to reduce morbidity and mortality from severe gastrointestinal and systemic infections. *E. coli* vaccine development efforts often target protein virulence factors that natively are extensively glycosylated, but this glycosylation is absent from recombinantly produced vaccine antigens. Human IgA responses to the conserved virulence factor YghJ have recently been shown to frequently target glycosylated epitopes. Here we evaluated to what extent anti-YghJ IgG responses also target glycosylated epitopes, longevity of these responses, and to what extent the responses correlated with the IgA responses.

**Methods:**

Multiplex bead flow cytometric immunoassays were used to evaluate changes in anti-YghJ IgG levels and glycosylation specificity in serum and antibody in lymphocyte supernatant (ALS) collected from 21 volunteers experimentally infected with enterotoxigenic *E. coli* (ETEC) strain TW10722.

**Results:**

Following infection, most volunteers had substantially increased anti-YghJ IgG levels both in serum and ALS. The proportion of serum anti-YghJ IgG that specifically targeted glycosylated epitopes increased from 0.10 (Interquartile range [IQR]: 0.07, 0.21) before to 0.17 (IQR: 0.11, 0.38) 10 days after dose ingestion before returning to pre-infection levels after 28 days. The glycosylation-specific proportions correlated between IgG and IgA for both serum and ALS.

**Conclusion:**

Our findings indicate that glycosylated epitopes are an important target for antibody immune responses and may play an important role in host immunity during the early phase of infection.

**Supplementary Information:**

The online version contains supplementary material available at 10.1186/s13099-025-00748-7.

## Background

Pathogenic *Escherichia coli* can cause a broad spectrum of illnesses in humans, ranging from diarrhea [1, 2] and urinary tract infection [3] to pneumonia, meningitis [4], and neonatal sepsis [5, 6]. *E. coli* infections can be recurrent and often difficult to treat [7], and treatment is further complicated by an increasing spread of multidrug resistance in the *E. coli* population [8, 9]. Antibodies generated in response to *E. coli* infections may protect against reinfections [10, 11], and this offers hope that vaccines against pathogenic *E. coli* can be developed.

During colonization, pathogenic *E. coli* often produce protein virulence factors that have undergone post-translational modifications [12], including mucinases like YghJ [13, 14], adhesins like EtpA [15], and fimbrial proteins [16]. Some of these modifications, like glycosylation, are believed to be important for proper functioning of the virulence factors and for evading cross-reactive immune responses [14, 15].

YghJ, also known as SslE, is secreted by most pathogenic and some non-pathogenic *E. coli* [17–20]. Although YghJ has several functions, including aiding biofilm formation [17, 18], it also acts like a virulence factor by degrading the mucus layer that protects epithelial surfaces [18, 21], allowing *E. coli* to colonize epithelial cells. Mouse studies have already shown that immunizing with YghJ may protect against sepsis [21] and that anti-YghJ antibodies may limit *E. coli* translocation and reduce colonization of the kidney and spleen [21]. For these reasons, YghJ has been targeted for vaccine development.

Native YghJ, especially from pathogenic *E. coli* is subject to extensive O-linked protein glycosylation [12–14], where glycans are added to serine and threonine residues exposed on the protein surface [12]. Glycosylated protein epitopes appear to be frequently targeted by the immune system during infection [22, 23].

Effective protection against gut microbial colonization is thought to mainly be attributed to the activities of secreted IgA [24, 25], but systemic IgG responses are also important [26–28], given they stop infections becoming systemic [29, 30] and help dampen inflammatory responses to the infections [31, 32]. Although IgA is relatively short-lived [33], IgG circulates for several months in the bloodstream, contributing to a long-lasting protection [34–36]. In humans, the majority of intestinal IgA and IgG antibodies are generated as a specific response to same antigenic stimulation [37], and thus may share similar antigen specificities. These IgA and IgG antibody populations, produced in response to an infection, can be studied in serum and in antibody in lymphocyte supernatants (ALS), which are created by in vitro culturing of the newly formed B-cells circulating in the blood around one week after infection onset [23, 38].

*E. coli* vaccine development efforts are mainly focused on subunit immunization with recombinant, non-glycosylated protein antigens, even when the natively expressed proteins are glycosylated. However, results from recent experimental infection studies have shown that serum and ALS IgA from volunteers experimentally infected with enterotoxigenic *E. coli* tend to target glycosylated epitopes of YghJ [22, 23]. Although it is unclear to what extent targeting glycosylated epitopes may have contributed to protecting the volunteers against infection or re-infection, these observations suggest that glycosylated epitopes are an important target of gut IgA responses. In this study, we aimed to examine to what extent anti-YghJ IgG responses also targeted glycosylated epitopes in these volunteers, whether these responses are long-lived, and to what extent they correlate with IgA. Given the fundamentally different roles and kinetics of IgA and IgG in mucosal and systemic immunity, these responses were analyzed in separate studies.

## Materials and methods

### Experimental ETEC infection study

The specimens and data used in this study were collected from a human experimental infection study carried out at the Division for Infectious Diseases ward at the Department of Medicine at Haukeland University Hospital, Bergen, Norway, between 2014 and 2018 [39]. In that study, 21 healthy volunteers aged between 19 and 28 years were infected with enterotoxigenic *E. coli* (ETEC) strain TW10722 (O115:H5; GenBank BioProject: PRJNA59745) that encodes ETEC colonization factors Coli Surface antigen 5 (CS5) and CS6 and the human variant of the heat-stable enterotoxin (STh) [40]. Doses ranging from 1 × 10^6^ to 1 × 10^10^ colony forming units (CFU) were given. Stool consistency was graded from 1 to 5, where grade 1 indicated firm/formed stools, grade 2 soft/formed, grade 3 viscous opaque liquid or semiliquid, grade 4 opaque liquid, and grade 5 clear/translucent liquid. Diarrhea was defined as ≥ 1 stool of grade ≥ 3 totalling ≥ 300 g, or ≥ 2 such stools totalling ≥ 200 g, within any 48-hour period during the first 120 h post-ingestion. Peak DNA shedding was measured by a TW10722-specific quantitative PCR assay targeting *wzy* gene allowing estimation of the relative abundance of TW10722 DNA in stool samples [41]. To clear the infection, ciprofloxacin was given 5 days after the dose ingestion or earlier if a volunteer developed moderate or severe diarrhea lasting for ≥ 24 h [39].

### Specimens

IgG from serum and ALS specimens were analyzed in this study. Serum specimens from all volunteers were collected 0 (before infection), 10, and 28 days after infection. For the last 12 volunteers enrolled, we also collected serum specimens after 6 and 12 months to enable analyses of long-term responses. Details of ALS specimen collection and preparation are given elsewhere [23]. Briefly, ALS was produced from peripheral blood mononuclear cells (PBMCs) isolated from venous blood specimens collected 0 (before infection) and 7 days after infection. Both specimen types were kept frozen at -80 °C until they were used for analyses.

### YghJ antigens

To facilitate comparison to results from previous studies, our analyses were based on the same batch of purified glycosylated and non-glycosylated YghJ (gYghJ and nYghJ, respectively) used previously [22, 23]. Briefly, gYghJ had been produced in wild-type TW10722 by inserting a 3xFLAG-coding sequence into the YghJ gene before culturing, while the non-glycosylated version was produced by cloning the 3xFLAG tagged YghJ gene into an expression vector and expressing it in *E. coli* strain MG1655Δ*hldE*, which lacks the ability to synthesize the heptose glycans needed by *E. coli* for protein glycosylation [42]. Later, both proteins were purified using affinity chromatography followed by dialysis. YghJ protein quality was tested by SDS-PAGE and quantification was done by using Bicinchoninic Acid (BCA) assay (Thermo Fisher Scientific, Waltham, MA). For characterization of glycosylation patterns, beta-elimination of O-linked glycans followed by Michael-addition of a phosphonic acid derivative (BEMAP) was used [22]. The antigens were stored frozen at -80 °C.

### Anti-YghJ IgG quantitation assay

Bead-based flow cytometry was used to quantify anti-YghJ IgG as described earlier for quantifying anti-YghJ IgA in serum [22] and ALS [23], except that MagPlex microspheres (Luminex Corp, Austin, TX) were used for all serum IgG analyses instead of Cyto-Plex beads (Thermo Fisher Scientific). For another study, initial tests using saliva specimens indicated that MagPlex beads gave lower background signals and allowed better washing steps during analyses and were therefore kept for serum IgG analyses. Bead-based multiplex assays were used instead of Enzyme-linked immunosorbent assay (ELISA) to minimize sample and antigen usage, and to allow simultaneous detection of different antibodies under standardized conditions, improving comparability and throughput.

Specimens were diluted in Assay buffer (PBS, 1% bovine serum albumin [BSA], and 0.05% Tween-20) and incubated with a 1:1 mix of 10,000 gYghJ- and nYghJ-coupled beads before washing in Assay buffer, labelled with 1:400-diluted R-phycoerythrin-labelled goat anti-Human IgG Fc secondary antibody (Jackson ImmunoResearch, West Grove, PA). ALS specimens were analysed on an LSR Fortessa flow cytometer (BD Life Sciences, Franklin Lakes, NJ) and serum specimens on Sony ID7000 spectral analyzer (Sony Biotechnology Inc. San Jose, CA). nYghJ-coupled beads were included in the assays for quality control purposes. As a measure of anti-YghJ IgG levels both in serum and ALS, we estimated the median fluorescence intensity (MFI) of the gYghJ-coupled beads by using FlowJo, version 10.4.2 (BD Biosciences). These values were normalized by comparing with MFI results from duplicate 10-fold dilution series of both a serum and an ALS specimen containing high anti-YghJ IgG levels, and where the highest MFI reading was set to 10,000 arbitrary units (AUs). Specimens with signal strength higher than the upper limit of standard curve were set to 10,000 AUs.

### Anti-YghJ IgG fold-change estimation

To estimate the fold-changes in anti-YghJ IgG antibody levels, we performed the anti-YghJ IgG quantitation assay with serum (1:100 diluted) and ALS (1:5 diluted). Anti-YghJ IgG levels from specimens collected after dose ingestion were divided by the anti-YghJ IgG levels from specimens collected immediately before dose ingestion. Specimens belonging to the same volunteer from different timepoints were analysed together. Volunteers mounting ≥ 2.0-fold increase in anti-YghJ IgG levels in serum and ALS were defined as responders.

### Glycosylation-specific proportion assay

We measured the degree to which anti-YghJ IgG responses specifically targeted glycosylated YghJ epitopes by performing the glycosylation-specific proportion (GSP) assay, the principle and methodology of which have been detailed earlier [23]. Briefly, by selectively depleting antibodies with excess soluble YghJ variants before bead-based detection, the GSP assay distinguishes glycosylation-specific responses and provides a functional readout of glycosylation-specific epitope targeting. All specimens were pre-treated with anti-IgA and -IgM agarose beads (Product no. A2691 and A9935, respectively, Sigma-Aldrich, St. Louis, MO) to deplete the specimens of IgA and IgM which could otherwise interfere with the GSP assay. We added 6 µL anti-IgA and 3 µL anti-IgM beads per microliter of 1:100 diluted serum, and 2.5 µL anti-IgA and 2.5 µL anti-IgM beads per microliter of 1:5 diluted ALS.

From the subsequent filtered supernatants, 50 µL were incubated with either 1 µg nYghJ, 1 µg gYghJ or 1 µL Assay buffer for 30 min shaken at room temperature to selectively hinder different types of anti-YghJ IgG in the specimens to bind to the MagPlex bead-coupled YghJ. The anti-YghJ IgG levels were subsequently estimated for all three aliquots as described for the anti-YghJ IgG quantitation assay, above. GSP for each volunteer was calculated by subtracting background level (IgG level after gYghJ treatment) from level of IgG targeting glycosylated YghJ epitopes (level after nYghJ treatment) and level of IgG targeting any YghJ epitopes (IgG level of untreated specimens) and then dividing the latter two. Serum specimens were included from all volunteers at all time points. To estimate change in GSP of serum anti-YghJ IgG, we divided the estimated post-infection anti-YghJ IgG proportions at each time point with the estimated pre-infection proportion. GSP analyses in ALS specimens were performed only on Day 7 as these specimens reflect immune response to a recent exposure.

### Statistical analysis

All statistical analyses were performed, and graphs were prepared in Prism version 10 (GraphPad Software, San Diego, CA), and p-values < 0.05 were considered to be statistically significant. Wilcoxon matched-pairs signed ranks test was used for intra-individual comparisons of IgG antibody levels and GSP values across timepoints, and to compare IgG vs. IgA GSP levels in paired specimens. Mann-Whitney U test was used to test for differences in anti-YghJ IgG antibody levels between volunteers who did and did not develop diarrhea. Pearson or Spearman correlation coefficients were used, as appropriate, to assess associations between antibody levels, GSP values with weight of stool and peak DNA shedding. Binary logistic regression analysis was performed to analyse relationship between inoculum dose and risk of developing diarrhea.

### Regulatory approval

The study (NCT02870751 at ClinicalTrials.gov) was approved by the Regional Committee for Medical and Health Research Ethics, Health Region West (REC-West; case number 2014/826). Study volunteers signed written informed consent and could leave at any time depending on their preference.

## Results

In the present study, we used bead-based multiplex flow cytometry to evaluate the fold changes and glycosylation specificity of anti-YghJ IgG in serum and ALS of 21 volunteers who had been experimentally infected with ETEC strain TW10722. Ten (48%) of the 21 volunteers developed diarrhea after dose ingestion. We found that increasing inoculum dose was significantly associated with risk of developing diarrhea (*p* = 0.031), with each 10-fold increase in dose conferring a 143% increase in odds of diarrhea.

### Anti-YghJ IgG antibody levels and fold changes

**Fig. 1:**
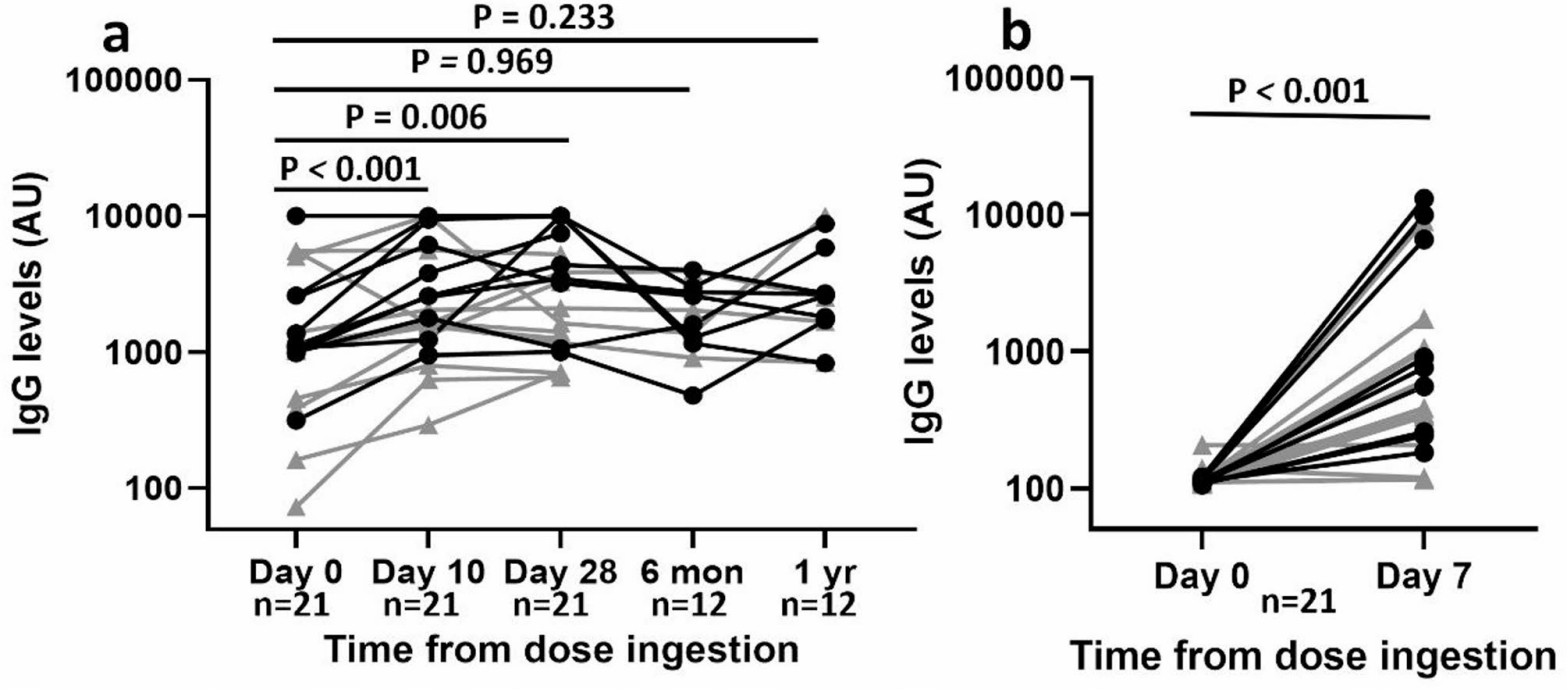
Changes in anti-YghJ IgG levels in serum and ALS. The logarithmically scaled lines show changes in anti-YghJ IgG in serum (**a**) and ALS (**b**) in 21 volunteers who had been experimentally infected with ETEC strain TW10722. Specimens with AUs > 10,000 are plotted as having AUs of 10,000. Black dots and grey triangles represent volunteers who did and did not develop diarrhea, respectively.

Serum anti-YghJ IgG levels in the 21 volunteers increased considerably from a median of 1067 (Interquartile range [IQR]: 986, 2513) AUs before dose ingestion to 1772 (IQR: 1327, 5571) AUs on Day 10, and 3197 (IQR: 1167, 5195) AUs on Day 28 (Fig. 1a). The median fold increase was 1.79 (IQR: 1.47, 3.01) and 1.54 (IQR: 1.14, 4.39), respectively. Ten (47%) of the 21 volunteers were responders, both when evaluated on Day 10 and Day 28. For the 12 volunteers who provided serum after 6 months and 1 year, the medians AUs at these time were similar to their pre-infection levels, with levels changing from 1244 (IQR: 1016, 3022) AUs on Day 0 to 1808 (IQR: 1243, 2812) AUs at 6 months and 2546 (IQR: 1698, 3485) AUs at 1 year. Different inoculum doses (10⁶–10¹⁰ CFU) did not significantly influence the magnitude of IgG responses in our cohort (Supplementary Fig. 1).

Anti-YghJ IgG levels in ALS increased 5.0 (IQR 2.4, 9.3) fold from a median of 113 (IQR: 109, 115) AUs prior to dose ingestion to a substantially higher median of 559 (IQR: 258, 1055) AUs after (Fig. 1b). Seventeen (81%) of the 21 volunteers were responders in the ALS analyses.

The volunteers who developed diarrhea appeared to have somewhat higher anti-YghJ IgG levels on Day 28, following the experimental infection than those who did not experience diarrhea. After adjusting for ingested dose, IgG levels on Day 28 remained borderline associated with diarrhea (Table 1). Similarly, the magnitude of serum anti-YghJ IgG responses correlated considerably to the weight of stool (in grams) on Day 28, but not on Day 10. No correlation was observed between serum anti-YghJ IgG responses and peak DNA shedding (Supplementary Fig. 2).

**Table 1:** Comparison of serum and ALS anti-YghJ IgG levels, in arbitrary units, between volunteers who did or did not develop diarrhea

Specimen and time point	Diarrhea Median (IQR) (*n*)	No diarrhea Median (IQR) (*n*)	*p*-value	Dose-adjusted *p*-value
**Serum**				
Day 0	1093 (997, 2227) (11)	1051 (420, 2968) (10)	0.689	na
Day 10	3191 (1962, 8589) (11)	1564 (1063, 1847) (10)	0.072	0.235
Day 28	5904 (3260, 10000) (11)	1406 (940, 2682) (10)	0.019	0.050
6 months	2085 (1243, 2812) (8)	1697 (1253, 2497) (4)	0.933	0.293
1 year	2621 (1788, 3485) (8)	2089 (1459, 9411) (4)	0.682	0.978
**ALS**				
Day 7*	835 (333, 5370) (11)	354 (271, 813) (10)	0.223	0.388

na = not applicable; *ALS IgG levels were negligible on Day 0 and were not included in these analyses

### Estimation of anti-YghJ IgG glycosylation-specific proportions and fold changes

**Fig. 2:**
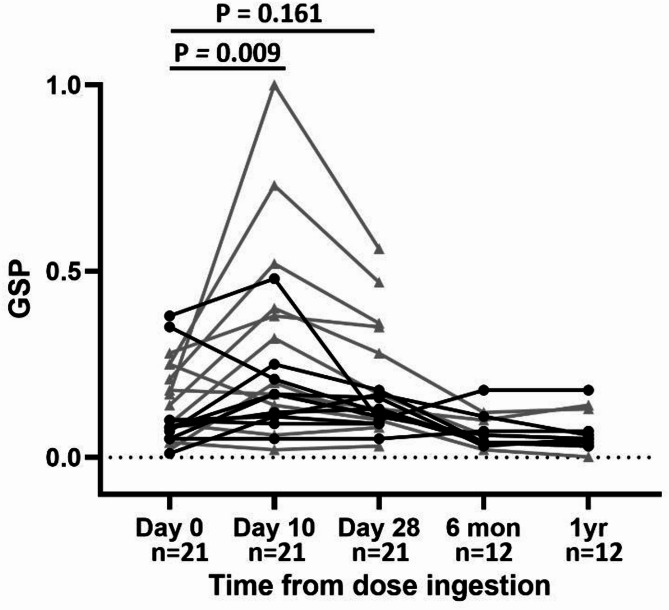
Changes in glycosylation-specific proportions (GSP) of anti-YghJ IgG in serum. The lines show changes in serum GSP of anti-YghJ IgG antibodies in 21 volunteers experimentally infected with ETEC strain TW10722. Black dots and grey triangles represent volunteers who did and did not develop diarrhea, respectively.

Serum specimens from all volunteers at all available timepoints were included in GSP analyses to assess the extent to which systemic IgG antibodies targeted glycosylated YghJ epitopes following the ETEC infection. The baseline median GSP on Day 0 was 0.10 (IQR: 0.07, 0.21; range: 0.01, 0.38) which increased notably to 0.17 (IQR: 0.11, 0.38; range: 0.02, 1.04) on Day 10 (Fig. 2). However, on Day 28 the GSP decreased again and was not clearly different from pre-infection level with a median of 0.13 (IQR: 0.10, 0.18; range: 0.03, 0.56). The median GSP levels appeared to return to pre-infection levels after 6 months (0.06 [IQR: 0.04, 0.08; range: 0.02, 0.18]) and 1 year (0.05 [IQR: 0.04, 0.08; range: 0.001, 0.18]) (Fig. 2). Administration of antibiotics at different time points for different volunteers did not interfere either with anti-YghJ IgG levels or GSP (Supplementary Fig. 3).

To obtain accurate GSP estimates, we excluded specimens that had anti-YghJ IgG levels < 400 AUs. The median ALS GSP of the Day 7 specimens from the remaining 11 responders was 0.11 (IQR: 0.06, 0.21; range: 0.01, 0.38). There was no clear difference in ALS and serum GSP between volunteers who did and did not develop diarrhea, with or without adjustment for inoculum (Table 2).

**Table 2:** Comparison of serum and ALS anti-YghJ IgG GSP between volunteers who did or did not develop diarrhea

Specimen and time point	Diarrhea Median (IQR) (*n*)	No diarrhea Median (IQR) (*n*)	*p*-value	Dose-adjusted *p*-value		
**Serum**						
Day 0	0.08 (0.05, 0.10) (11)	0.17 (0.09, 0.23) (10)	0.305	na		
Day 10	0.15 (0.11, 0.20) (11)	0.32 (0.16, 0.46) (10)	0.146	0.140		
Day 28	0.12 (0.10, 0.15) (11)	0.17 (0.10, 0.36) (10)	0.215	0.057		
6 months	0.05 (0.04, 0.08) (8)	0.08 (0.05, 0.10) (4)	0.832	0.248		
1 year	0.05 (0.04, 0.06) (8)	0.09 (0.04, 0.13) (4)	0.773	0.162		
**ALS**						
Day 7*	0.13 (0.06, 0.21) (4)	0.11 (0.08, 0.18) (7)	0.927	0.175		

na = not applicable; *ALS GSP analyses were not performed on Day 10

### Correlation between anti-YghJ IgG and IgA responses in targeting glycosylated epitopes

**Fig. 3:**
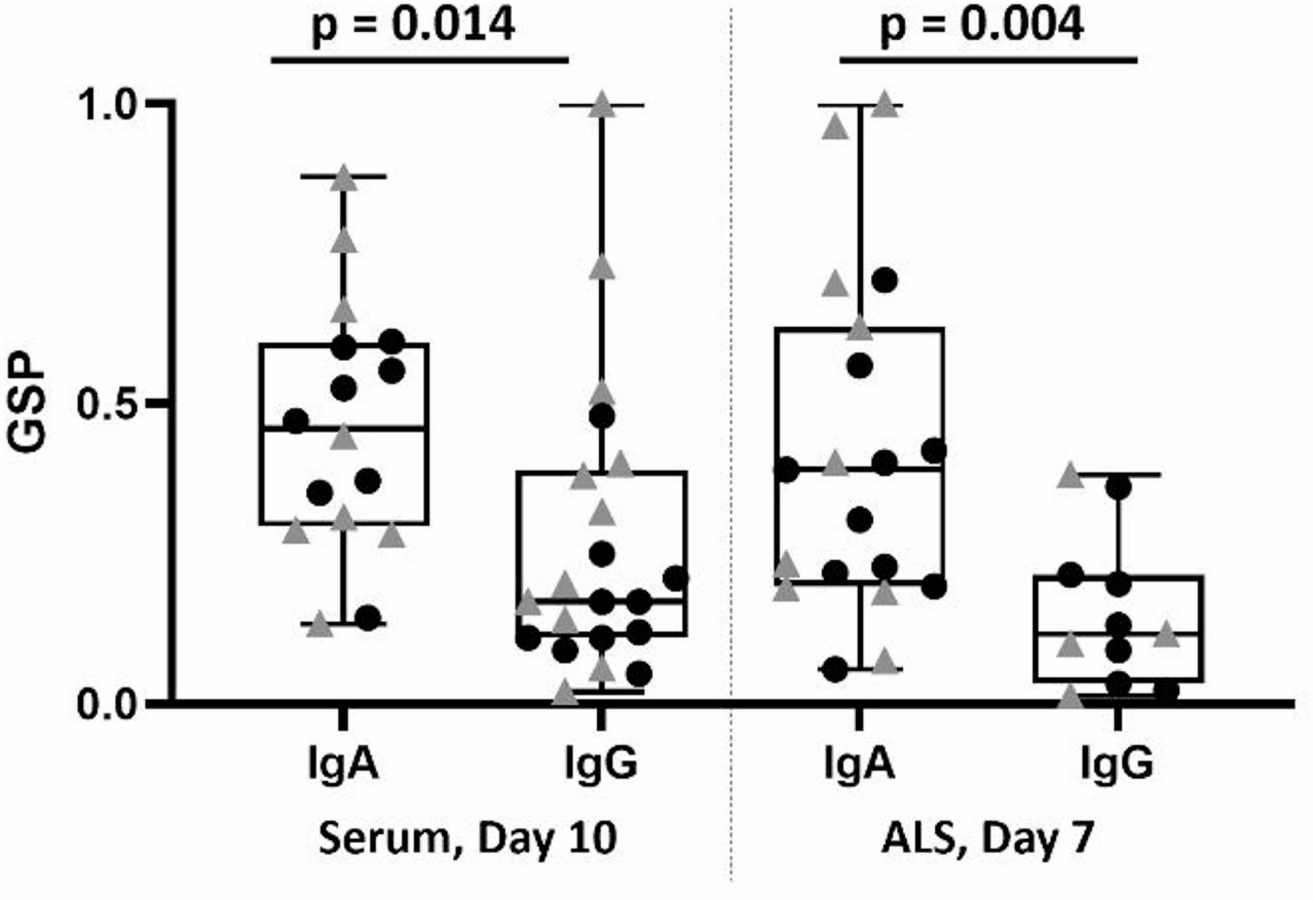
Boxplots of the glycosylation-specific proportions (GSP) of anti-YghJ IgA and -IgG from serum and ALS specimens. Horizontal lines indicate the median, interquartile range, and range. Black dots and grey triangles represent volunteers who did and did not develop diarrhea, respectively.

Previously, the median GSP of anti-YghJ IgA in serum and ALS from these volunteers were found to be 0.45 (IQR: 0.30, 0.59; range: 0.13, 0.88; *n* = 16) and 0.39 (IQR: 0.20, 0.59; range: 0.05, 1.04; *n* = 19), respectively [22]. These GSP estimates were 1.70 and 2.70 times higher than what we found in the present study for serum and ALS IgG (Fig. 3).

**Fig. 4:**
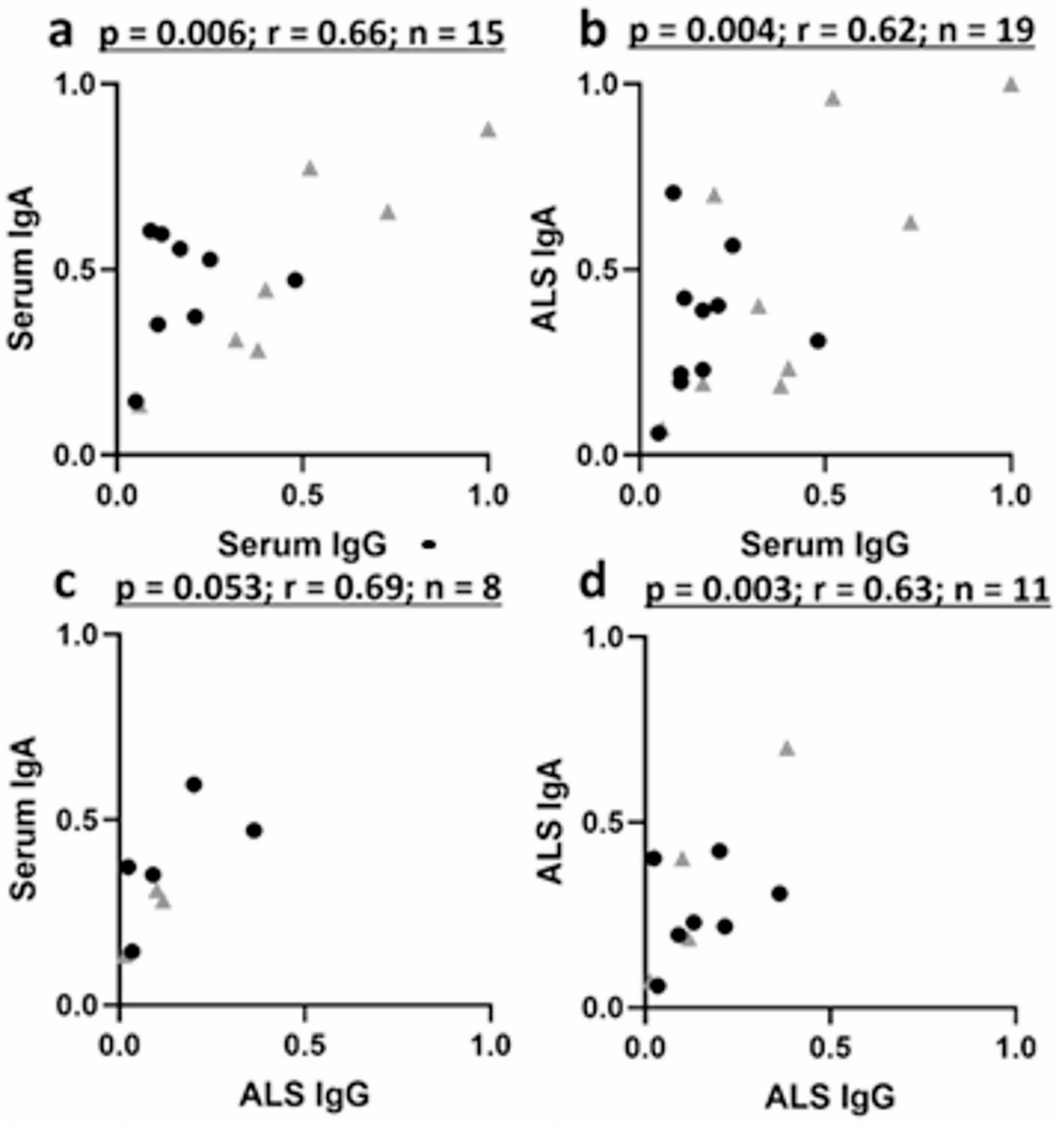
Correlation of anti-YghJ IgG and anti-YghJ IgA glycosylation specific proportions (GSP) in serum and ALS. The r denotes the Pearson’s correlation coefficient and p the corresponding p-values. Black dots and grey triangles represent volunteers who did and did not develop diarrhea, respectively.

GSP values were strongly correlated across isotypes and compartments: serum IgA with IgG, serum IgG with ALS IgA, and ALS IgA with ALS IgG (Fig. 4). The correlation between serum IgA GSP on Day 10 and ALS IgA GSP on Day 7 post-infection with serum IgG GSP remained strong also on Day 28 (Supplementary Fig. 4).

## Discussion

In this study we characterized systemic IgG responses to a natively glycosylated *E. coli* antigen, YghJ, in human volunteers who had been experimentally infected with ETEC and tested to what extent the anti-YghJ IgG antibody responses targeted glycosylated epitopes. The results reveal a temporal increase in antibody reactivity to glycosylated YghJ, highlighting how an *E. coli* infection may shape glycosylated epitope recognition.

Recently, there has been an increased recognition of the role of IgG in intestinal immunity [30, 31]. In particular, it has lately been shown that serum IgG produced in response to conserved antigens from commensal gut bacteria may help confer cross-protection against other gut pathogens [29, 32, 43]. A pool of protective IgG antibodies hence exists in circulation against antigens from a wide variety of pathogens [30, 31].

Targeting antigens shared by both pathogenic and commensal strains as vaccine candidates is not uncommon and does not necessarily diminish their protective potential. For example, the conserved heparin-binding protein, also known as NHBA, present in both pathogenic and non-pathogenic *Neisseria* strains, is a key component of the licensed 4CMenB (Bexsero^®^) vaccine and has been shown to elicit protective immunity in humans [44, 45]. YghJ is also a conserved mucinase among commensal and pathogenic *E. coli* [20, 46], so it was not unexpected to find substantial serum baseline IgG levels against YghJ in several volunteers. However, we did not observe any clear association between pre-infection anti-YghJ IgG levels in serum or ALS and experiencing a diarrheal episode.

On the other hand, after infection, anti-YghJ IgG levels were notably higher in volunteers who developed diarrhea as compared to the non-diarheal group on Day 28. Although no direct association was found between inoculum dose and IgG levels, the observed link between higher doses and increased diarrhea rates, and between diarrhea and elevated IgG, suggests that clinical severity may modulate systemic antibody responses. This is consistent with prior findings that substantial bacterial proliferation in the gut, even by non-invasive pathogens as ETEC, can result in systemic inflammation, mucosal injury [47, 48], and diarrhea [41], potentially driving the elevated IgG levels observed in volunteers with diarrhea.

Conversely, changes in the GSP of anti-YghJ IgG did not seem to be associated with diarrhea, suggesting that the antibody fraction targeting glycosylated epitopes is independent of the underlying conditions that induces an antibody response.

Antibody levels in ALS specimens reflect antigen-specific response to a recent exposure [38, 49]. Negligible ALS anti-YghJ IgG levels on Day 0 indicate that the relatively high levels of pre-infection YghJ-specific IgG antibodies found in serum are a part of IgG pre-existing in circulation and not a response to a recent exposure. This lack of B-cell activation, despite YghJ being encoded by commensal strains, suggests that expression of YghJ alone is insufficient to trigger immune priming. Rather, its immunogenicity likely depends on infection-associated factors such as antigen abundance, secretion into the mucosal environment, specific glycosylation patterns, and the presence of local inflammation, all of which can enhance immune visibility [20, 21, 50, 51].

A low serum GSP at baseline therefore shows that, in the absence of recent or active infection, anti-YghJ IgG antibodies mainly target non-glycosylated epitopes on YghJ from TW10722. However, we found a considerable increase in GSP of serum anti-YghJ IgG immediately after infection, but the GSP levels started to return to pre-infection levels 28 days afterwards, even though anti-YghJ IgG levels were still significantly high at this timepoint. It could be because once the infection starts, it will result in high levels of secreted glycosylated YghJ that will result in high GSP. But a decrease after 28 days indicates that glycosylated epitopes may be subject to a broader, but short-lived response that wanes during the antibody maturation process.

During gut infections, IgA and IgG antibodies work through distinct mechanisms and in discrete anatomical compartments to regulate intestinal immunity [43], where short-lived and mucosal IgA may entrap gut microbes, and IgG may prevent systemic infection [35, 52]. In serum, both these antibodies have partly originated from antigen activated B cells in the gut mucosa [53, 54]. A correlation between glycosylation-specific proportions of systemic IgG and IgA suggests that although plasmablasts secreting IgG and IgA antibodies do not originate from common B cell precursors, they have been induced by the same antigen exposure [37]. These findings also indicate that during early phase of adaptive immune response a coherent IgG and IgA response was generated that targeted glycosylated epitopes.

### Limitations

Volunteers who developed diarrhea were given antibiotics earlier than the volunteers who did not, thereby increasing the antigen exposure duration for the non-diarrheal group. However, timing of antibiotic treatment did not significantly influence systemic IgG responses, suggesting that even brief antigen exposure was sufficient to elicit measurable responses in most volunteers. Antibiotic treatment nonetheless shortened the duration of the experimental infection in all volunteers and may have influenced the antibody development and maturation.

Neither precise identification of which glycosylated and non-glycosylated epitopes were targeted by IgG, nor the avidity of these antibodies were analysed in this study. Further investigations may give insight into whether IgA and IgG targeted the same epitopes and whether glycosylation-specific antibodies show high avidity compared to non-glycosylation-specific antibodies, as well as specific effector functions of glycosylation-specific antibodies.

## Conclusions

Our data show that volunteers’ exposure to pathogenic *E. coli* through an ETEC gastroenteritis induced a substantial anti-YghJ IgG response in serum and ALS. We provide data showing a transient, but considerably increased proportion of anti-YghJ IgG antibodies in serum specifically targeting glycosylated epitopes of YghJ after infection.

We also found a correlation between proportions of anti-YghJ IgG and IgA antibodies that target glycosylated epitopes, both in serum and ALS, in the early phase of the adaptive immune response.

These findings indicate that carbohydrate moieties form significant parts of immunogenic epitopes of YghJ. They may play an important role in the broad antibody response to tackle acute infection and modulate host immunity towards protection against reinfection. Although further studies are needed to determine whether our findings can be generalized to other glycosylated *E. coli* antigens, the observed change in glycosylation specificity of the systemic IgG response over time suggests that glycosylation of vaccine antigens may be important to induce an antibody response akin to that developed after natural infection.

## Supplementary Information

Below is the link to the electronic supplementary material.

Supplementary Material 1

## Data Availability

No datasets were generated or analysed during the current study.
