# Supplementary material for: Systemic IgG responses to glycosylated mucinase YghJ after experimental enterotoxigenic Escherichia coli infection
Source: Gut Pathog. 2025 Sep 25;17:70. doi: 10.1186/s13099-025-00748-7 (PMC12465375; doi:10.1186/s13099-025-00748-7)
Supplement: Supplementary file 1 — Supplementary Material 1 [file 13099_2025_748_MOESM1_ESM.docx]

**Systemic IgG responses to glycosylated mucinase YghJ after experimental enterotoxigenic *Escherichia coli* infection**

**Saman Riaz ^1^, Hans Steinsland ^2,3^, Anders Boysen^4^, Kurt Hanevik ^1,5^***

^1^ Department of Clinical Science, University of Bergen, Bergen, Norway

^2^ Centre for Intervention Science in Maternal and Child Health (CISMAC), Centre for International Health, Department of Global Public Health and Primary Care, University of Bergen, Bergen, Norway

^3^ Department of Biomedicine, University of Bergen, Bergen, Norway

^4^ GlyProVac ApS, Rørhatten 4, Odense, Denmark

^5^ National Centre for Tropical Infectious Diseases, Department of Medicine, Haukeland University Hospital, Bergen, Norway

***** Correspondence: kurt.hanevik@uib.no; Tel.: +47 93856690; ORCID: 0000-0002-1466-2326

**Supplementary data**

**Figure 1. Correlation between the dose of inoculum and anti-YghJ IgG antibody levels (a) and GSP (b)**

**
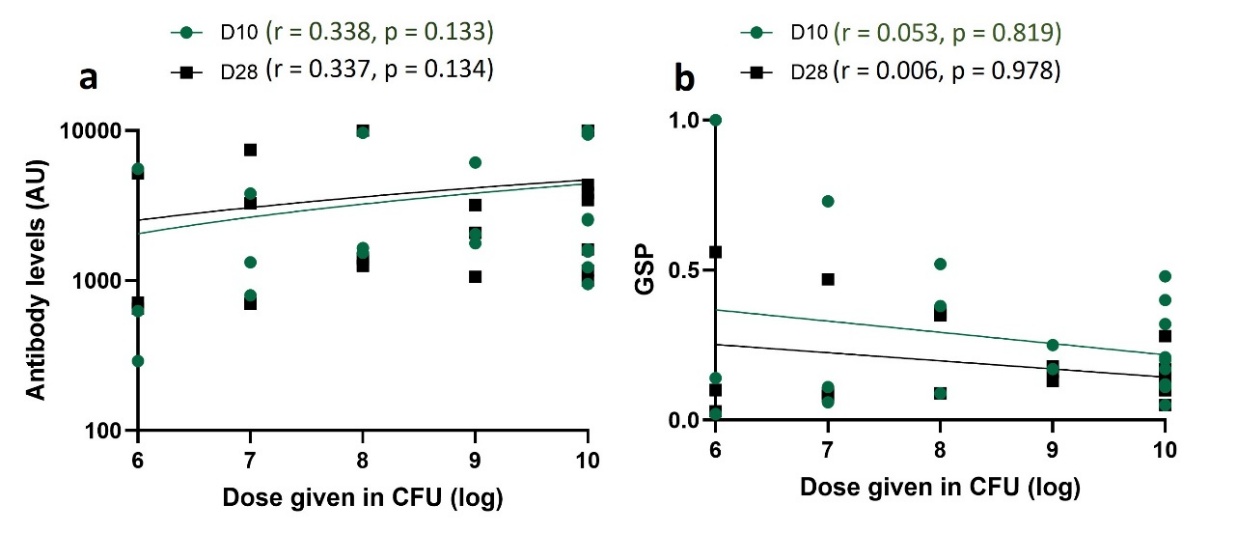
**

The r denotes the Spearman’s correlation coefficient and p the corresponding p-values. Green dots and black squares dots represent volunteers on Day 10 and Day 28, respectively.

**Figure 2. Correlation between peak DNA shedding and anti-YghJ IgG antibody levels (a) and GSP (b), and total weight of stool and anti-YghJ IgG antibody levels (c) and GSP (d)**

**
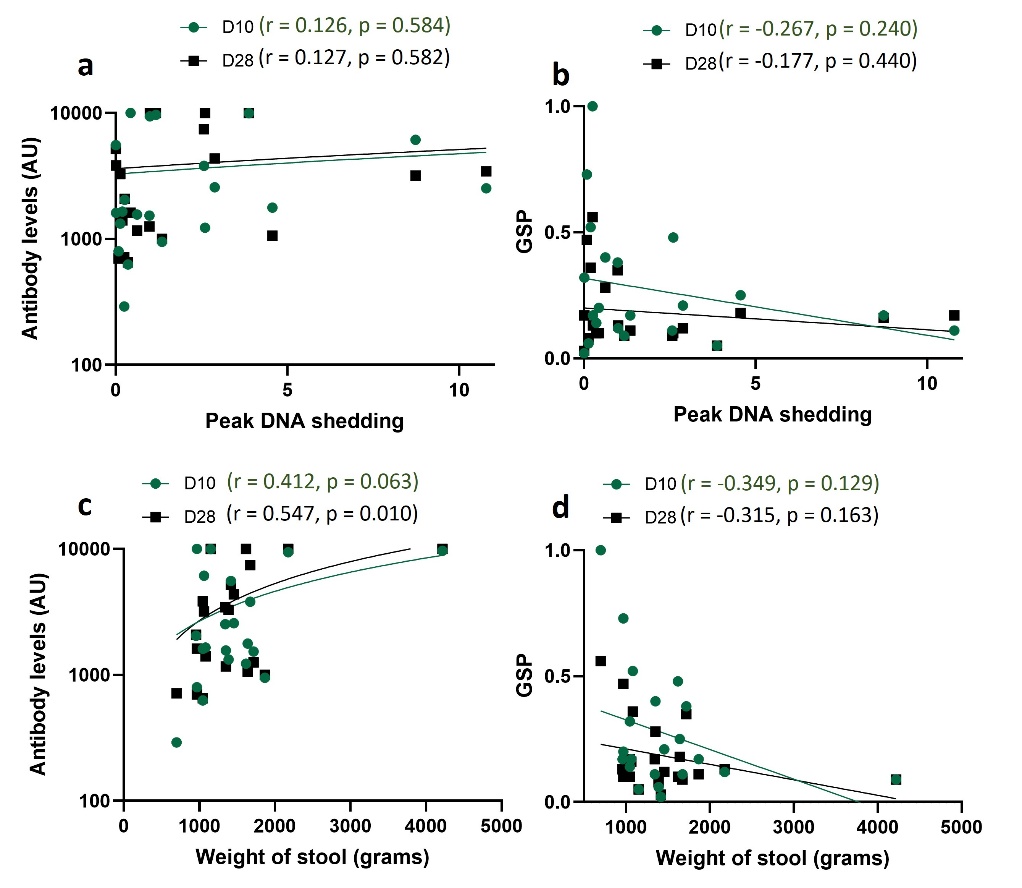
**

The r denotes the Pearson’s correlation coefficient and p the corresponding p-values, and n is 21. Green dots and black squares dots represent volunteers on Day 10 and Day 28, respectively.

**Figure 3. Correlation between time of antibiotic administration and anti-YghJ IgG antibody levels (a) and GSP (b)**

**
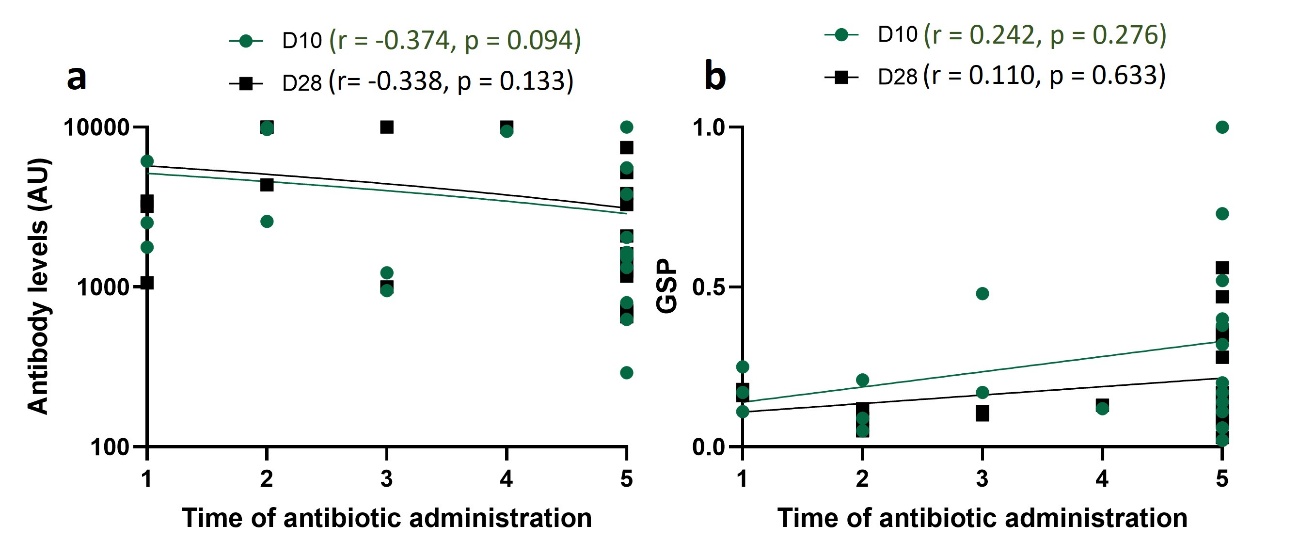
**

The r denotes the Spearman’s correlation coefficient and p the corresponding p-values. Green dots and black squares dots represent volunteers on Day 10 and Day 28, respectively.

**Figure 4. Correlation of anti-YghJ IgG and anti-YghJ IgA glycosylation specific proportions (GSP) in serum (a) and ALS (b)**


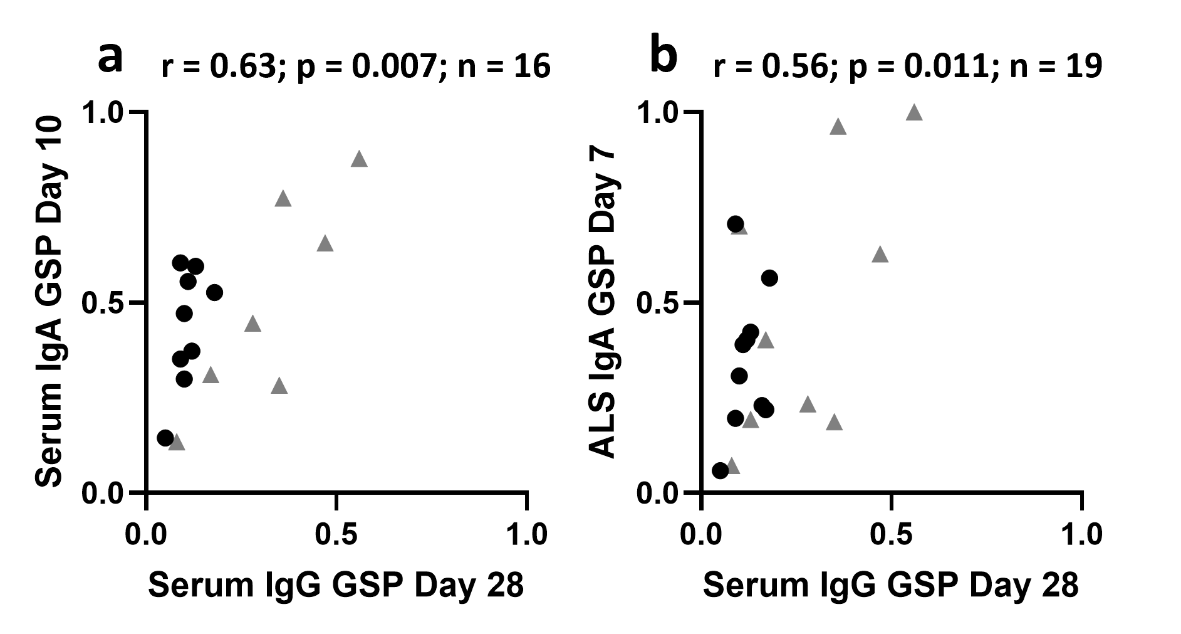


The r denotes the Pearson’s correlation coefficient and p the corresponding p-values. Black dots and grey triangles represent volunteers who did and did not develop diarrhea, respectively.
